# A Single Liver Metastasis from Pleural Biphasic Mesothelioma

**DOI:** 10.3390/diagnostics10080555

**Published:** 2020-08-04

**Authors:** Andrea Marzullo, Gabriella Serio, Federica Pezzuto, Francesco Fortarezza, Gerardo Cazzato, Concetta Caporusso, Teresa Lettini, Domenica Cavone, Maria Celeste Delfino, Luigi Vimercati

**Affiliations:** 1 Department of Emergency and Organ Transplantation, Pathology Division, University of Bari, 70124 Bari, Italy; andrea.marullo@uniba.it (A.M.); gerycazzato@hotmail.it (G.C.); kcaporusso.c@libero.it (C.C.); lettinit@yahoo.com (T.L.); 2 Department of Cardiac, Thoracic, Vascular Sciences and Public Health, University of Padova, 35121 Padova, Italy; federica.pezzuto@phd.unipd.it (F.P.); francescofortarezza.md@gmail.com (F.F.); 3 Department of Interdisciplinary Medicine, Occupational Health Division, University of Bari, 70124 Bari, Italy; domenica.cavone@uniba.it (D.C.); mcelestedelfino@gmail.com (M.C.D.); luigi.vimercati@uniba.it (L.V.)

**Keywords:** autopsy, malignant pleural mesothelioma, liver, metastasis, asbestos exposure

## Abstract

Virtually any malignancy can metastasize to the liver. Large solitary metastases are rare and can be difficult to distinguish from primary tumors. Malignant mesothelioma is often considered as a locally invasive cancer but tumor dissemination to extra-thoracic sites is possible, and the liver can be involved. Herein, we present a rare case of pleural mesothelioma with a solitary large liver metastasis diagnosed postmortem in a ninety-two-year-old man with 35 years of exposure to asbestos. Results of immunohistochemical staining of the pleural and liver tumor were similar, both positive for low-molecular weight keratins, calretinin, vimentin, and podoplanin, and negative for Claudin-4, TTF1, CEA, BerEP4, CK7, CK19, CK20, BAP1, Hep Par1, p40, and WT1. Fluorescent in-situ hybridization (FISH) for p16/CDKN2A was also performed and a homozygous deletion was detected in both tumors, supporting the diagnosis of mesothelioma. Reporting this case, we would like to point out that extra-thoracic dissemination from pleural mesothelioma, even if exceptional, can occur. In cases where differential diagnoses are challenging, the value of ancillary techniques and a practical approach to diagnostic work-up is of primary importance.

**Figure 1:**
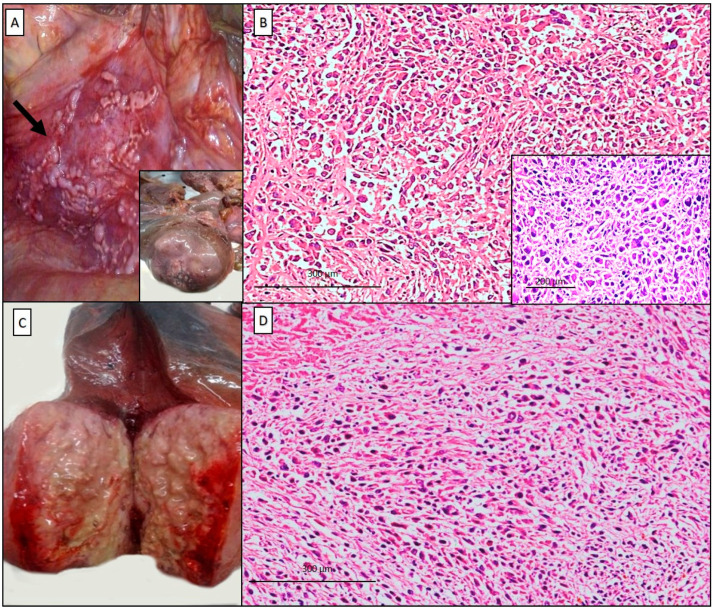
Autopsy was performed in a ninety-two-year-old male at the request of his son, on the suspicion of an occupational disease of legal concern. The patient died at home; in the last month he had presented weight loss, coughing, chest pain, fever, and general malaise. Chest CT-scan revealed an intrathoracic mass in the right upper lobe measuring 6.7 × 6.5 cm, of probable pleural origin; numerous bilateral pleural plaques were also detected. The patient, bedridden due to the serious general conditions, was not subjected to further investigations and died in few weeks. Gross examination revealed multiple nodular bilateral scleral hyaline plaques of the costal, parietal, and diaphragmatic pleura (**A**, arrow). At the upper lobe of the right lung, a nodular mass, 9 cm in diameter, adhered to the thoracic surface (**A**, inset). The right pleural surface was thickened (1 cm). At histology, the thoracic mass was characterized by a proliferation of epithelioid malignant cells showing a solid pattern, vesicular round nuclei with small nucleoli, and an admixture of pleomorphic epithelioid and spindle cells (**B**, hematoxylin and eosin, scale bar: 300 µm; inset: pleomorphic features). The right lobe of the liver was entirely occupied by a partially necrotic mass, 10 cm in diameter (**C**), and the liver cancer showed prevalent large necrosis foci and neoplastic aggregates with similar microscopic features (**D**, hematoxylin and eosin, scale bar: 300 µm).

**Figure 2:**
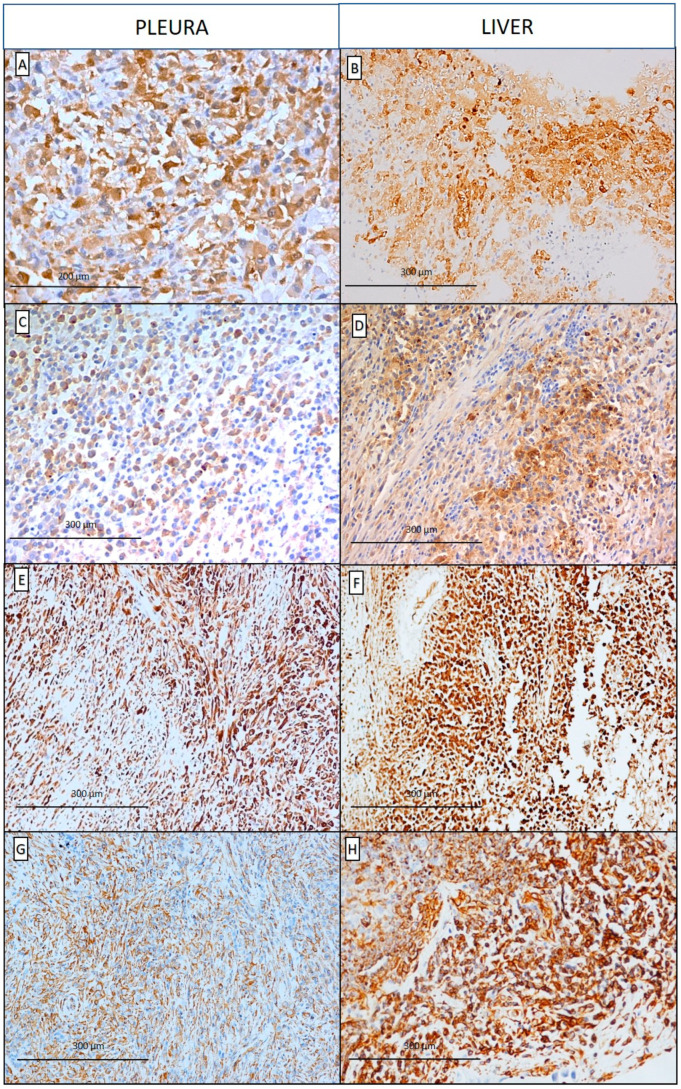
According to the recent guidelines [1,2] a panel of immunohistochemistry antibodies was used in both tumors, eliciting positive reactions to: calretinin (**A**, scale bar: 200 µm; **B**, scale bar: 300 µm), podoplanin (D2-40) (**C**,**D**, scale bar: 300 µm), CK5/6 (**E**,**F**, scale bar: 300 µm), and vimentin (**G**,**H**, scale bar: 300 µm), and negative reactions to CEA, TTF-1, p40, CD34, HMB45, Melan-A, CK7, CK19, CK20, PAX8, FLI-1, and BAP1 (using lymphocytes as positive control). Immunostaining for WT-1 was also performed, resulting negative in both lung and liver.

**Figure 3:**
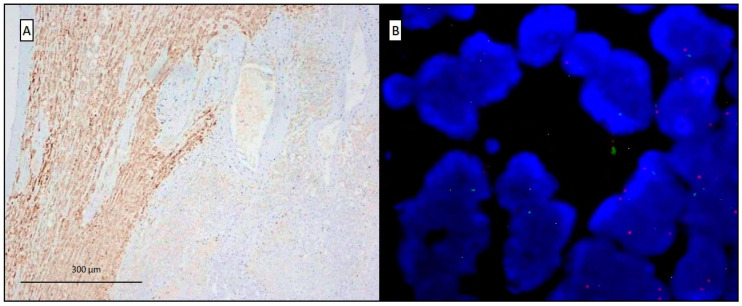
In the liver, Hep Par-1 (OCH1E5) was negative, marking only the hepatocytes and some necrotic debris (**A**, scale bar: 300 µm), and fluorescent in-situ hybridization (FISH) was performed for the *CDKN2A*/p16 gene, revealing the homozygous deletion (more than 50%) (**B**). The same results were obtained with examination of the pleural mass. Based on the subject’s clinical history (featuring 35 years of occupational exposure to asbestos on board ships), the radiological data (reporting a mass arising from the pleural surface), and all morphological, immunohistochemical, and molecular analyses, the final diagnosis was judged to be most in favor of a malignant mesothelioma. The detection of several fibrotic pleural plaques, the coexistence of intermingled epithelioid (with pleomorphic elements) and sarcomatoid areas, and the abundance of necrosis within the liver tumor led to the diagnosis of a malignant biphasic pleural mesothelioma with a single liver metastasis. Malignant pleural mesothelioma (MPM) is a rare incurable cancer related to occupational or environmental asbestos exposure [3,4]. Generally, the tumor is considered to be a locally aggressive cancer and distant metastases are often not considered a common feature. Intrathoracic mediastinal structures and the contralateral hemithorax are the sites most commonly involved. In the literature, the extra thoracic metastases most frequently reported, with different percentages are peritoneum, intestine, liver, spleen, bone, and brain [5,6,7]. Liver is one of the organs where metastases are more common than primary tumors. Solitary metastases occur in about 6% of all metastases to the liver. Solitary large masses measuring more than 5 cm in diameter are rarer and can be clinically difficult to distinguish from primary cancer [7,8]. Moreover, primary intrahepatic mesotheliomas arising from the mesothelial cell layer over the Glisson’s capsule are also reported, rarely associated to asbestos exposure [9]. Diagnosis of these rare tumors can be very challenging. Using a large panel of immunohistochemistry antibodies and molecular analyses can help pathologists to diagnose this rare malignancy. The epithelioid type is often easy to identify and is associated with the best prognosis among all histotypes [10]. Some ambiguous entities exist, such as the transitional mesothelioma pattern [11] and pleomorphic type [12], more likely affected by an aggressive behavior and sharing some difficulties in the diagnostic approach [13]. Both these differential diagnoses were considered in our case. Indeed, some epithelioid cells showed pleomorphic features, with nuclear enlargement, hyperchromasia, large nucleoli, and rare multinucleation [12]. However, the examination of the whole tumors revealed the coexistence of two different and distinguishable components, epithelioid and sarcomatoid, thus orienting toward a biphasic form. Immunohistochemical and molecular analyses could also be helpful in these challenging cases. Nevertheless, the sensitivity and specificity of some antibodies may be variable. Specifically, in our case, the WT1 negativity was not an unexpected finding, as this is reported to be expressed in less than half of high-grade mesotheliomas [14,15]. Similarly, other marker expressions can be lost in the sarcomatoid component, such as calretinin and CK5/6, whose sensitivity ranged from 10 to 60% and 13 to 29%, respectively [14]. Conversely, podoplanin is frequently detected in high-grade tumors (with a sensitivity of up to 90%), but its specificity is low [14]. In our patient, the combination of different immunohistochemical markers, together with the detection of p16/CDKN2A deletions, most frequently reported in mesotheliomas [1,16], permitted a confident identification of the mesothelial lineage of the neoplastic cells. In conclusion, our case confirms the value of ancillary techniques and of a practical approach to the diagnostic work-up for diagnosing mesothelioma, particularly in challenging cases. Malignant pleural mesothelioma should be considered a neoplasm with an extra-thoracic metastatic capacity like most cancers, and this aspect should be taken into account in clinical practice.
